# A Scoping Review of the Quality and the Design of Evaluations of Mobile Health, Telehealth, Smart Pump and Monitoring Technologies Performed in a Pharmacy-Related Setting

**DOI:** 10.3389/fphar.2018.00678

**Published:** 2018-07-26

**Authors:** Darrin Baines, Imandeep K. Gahir, Afthab Hussain, Amir J. Khan, Philip Schneider, Syed S. Hasan, Zaheer-Ud-Din Babar

**Affiliations:** ^1^Department of Accounting, Finance and Economics, Bournemouth University, Poole, United Kingdom; ^2^Faculty of Health and Life Sciences, Coventry University, Coventry, United Kingdom; ^3^Community, Environment and Policy Department, University of Arizona College of Pharmacy, University of Arizona, Tucson, AZ, United States; ^4^Pharmaceutical Policy and Practice Research Centre, University of Huddersfield, Huddersfield, United Kingdom

**Keywords:** pharmacy, pharmaceutical care, mobile health, telehealth, smart pumps, monitoring technologies

## Abstract

**Background:** There is currently a need for high quality evaluations of new mobile health, telehealth, smart pump and monitoring technologies undertaken in a pharmacy-related setting. We aim to evaluate the use of these monitoring technologies performed in this setting.

**Methods:** A systematic searching of English articles that examined the quality and the design of technologies conducted in pharmacy-related facilities was performed using the following databases: MEDLINE and Cumulative index to Nursing and Allied Health Literature (CINAHL) to identify original studies examining the quality and the design of technologies and published in peer-reviewed journals. Extraction of articles and quality assessment of included articles were performed independently by two authors. Quality scores over 75% are classed as being acceptable using a “relatively conservative” quality benchmark. Scores over 55% are included using a “relatively liberal” cut-off point.

**Results:** Screening resulted in the selection of 40 formal evaluations. A substantial number of studies (32, 80.00%) were performed in the United States, quantitative in approach (33, 82.50%) and retrospective cohort (24, 60.00%) in study design. The most common pharmacy-related settings were: 22 primary care (55.00%); 10 hospital pharmacy (25.00%); 7 community pharmacy (17.50%); one primary care and hospital pharmacy (2.50%). The majority of the evaluations (33, 82.50%) reported clinical outcomes, six (15.00%) measured clinical and economic outcomes, and one (2.50%) economic only. Twelve (30.00%) quantitative studies and no qualitative study met objective criteria for “relatively conservative” quality. Using a lower “relatively liberal” benchmark, 27 quantitative (81.82%) and four qualitative (57.41%) studies met the lower quality criterion.

**Conclusion:** Worldwide, few evaluations of mobile health, telehealth, smart pump and monitoring technologies in pharmacy-related setting have been published.Their quality is often below the standard necessary for inclusion in a systematic review mainly due to inadequate study design.

## Introduction

Pharmacy is an evidence-based profession (Toklu, 2015). Pharmacists are generally unwilling to recommend unproven health technologies, especially when their adoption could have negative unanticipated consequences (Sun and Qu, 2015). Evidence of effectiveness and cost-effectiveness is vitally important for decision-makers (Dawoud and Baines, 2017). New technologies often face adoption problems due to a scarcity of suitable evidence (Van Gemert-Pijnen et al., 2011). A deficiency of scientific studies is a widespread problem in all areas of healthcare provision, including pharmacy (Chaudhry et al., 2006; Atienza et al., 2010; Black et al., 2011; FIP/WHO, 2011). There is a growing gap between the rate of technological advance and the production of supporting evaluations. For instance, there are now over 165,000 health-related apps available on the main smartphone operating systems (The Economist, 2016). Few are launched with accompanying high-quality, peer-reviewed evidence demonstrating their effectiveness and/or cost-effectiveness (Steinhubl et al., 2015).

Technological innovation is important in all industries (Burns and Stalker, 1961). For a pharmacy workforce that has been relatively static for many decades, disruptive technological change (a change that creates a new system and eventually disrupts an existing system) could create a “technology shock” (Spinks et al., 2016). Disrupting technologies can enhance adaptability and flexibility, helping pharmacists to identify and to solve unfamiliar problems in unfamiliar situations (Woods et al., 2015). For instance, the profession could use smart packaging to extend its surveillance of medicines taking (Darkins et al., 2008; Sparks, 2011). As the widespread adoption of robotics and bar-code technologies may soon reduce the number of pharmacists involved in dispensing, pharmaceutical care may be the way ahead (Hepler and Strand, 1990; Baines, 2015). In response, we label (non-dispensing) technologies that enable pharmacists to improve patient health and quality of life with the name of “pharmaceutical care technologies.” We suggest that mobile health (general term for the use of mobile phones and other wireless technology to educate consumers about preventive health care services), telehealth (the distribution of health-related services and information via electronic information and telecommunication technologies), smart pump (programmable infusion device for controlling and administering intravenous medicines) and monitoring technologies (e.g., microchip-containing tablet blister for adherence monitoring) are amongst this group of innovations.

With a deficit of available evidence, pharmacists do not always have the information required to make rational technology adoption decisions (Gregorio et al., 2013). Even when evaluation studies are published, many are poorly designed and their low quality can misguide decision-makers (Shuren and Califf, 2016). To improve this situation, the constant production of well-designed, high-quality evaluations is required. The first step in improving the current situation is to evaluate the current state of the technology appraisal literature that focuses on pharmacy practice. This paper is a scoping review of the quality and the design of evaluations of mobile health, telehealth, smart pump and monitoring technologies performed in a pharmacy-related setting. To our knowledge, no review has yet analyzed the quality and the design of peer-reviewed evaluations of these technologies in this setting. The rationale of this review is to help fill that gap.

## Methods

### Scope of review: eligibility criteria

A scoping review was undertaken to identify evaluations of mobile health, telehealth, smart pumps and monitoring technologies based upon the classification of technologies identified by Goundrey-Smith (2013, 2014) The review assesses the quality and the design of the identified studies. We limited our search to studies written in English and did not restrict them according to country. Studies had to measure either clinical or economic outcomes, or both. The studies had to fall into our defined technology categories. All study designs (including trials, protocols) were included in the search process. However, only evaluation studies were included in the final analysis.

This scoping review examined the quality and the design of studies published in peer-reviewed journals that report scientific evaluations of mobile health, telehealth, smart pump and monitoring technologies tested in a pharmacy-related setting. This paper is written from the point of view of pharmacy practice research. The quality of the evaluations identified is judged in terms of a composite quality score developed by Kmet et al. (2004) which assesses study design in terms of structure, methodology and outcomes measured. The main objectives of our review are to: (i) identify all relevant studies published in a peer-review journal that evaluate pharmaceutical care technologies; (ii) evaluate the quality of this work; (iii) identify the study designs and the clinical/economic outcomes used; (iv) examine the link between study design and evaluation quality; (v) outline the limitations of our review. To achieve these objectives, we perform the following interlinked tasks. First, we outline our review methods, including inclusion criteria and our approach to quality scoring. Second, our results are presented, including data on quality and design. Third, we discuss the main findings of the review. Finally, we outline the limitations of our work.

### Definitions

Hepler and Strand (1990) defined “pharmaceutical care” as the “responsible provision of drug therapy for the purpose of achieving definite outcomes that improve a patient's quality of life.” Subsequently, the board of the Pharmaceutical Care Network Europe (Allemann et al., 2014) suggested that “pharmaceutical care” may be defined as the “pharmacist's contribution to the care of individuals to optimize medicines use and improve health outcomes.” Combining both definitions, we define “pharmaceutical care technologies” as “technologies that enable pharmacists to optimize medicines use and to enhance patient health and quality of life.”

### Information sources

The following databases were searched: MEDLINE and Cumulative index to Nursing and Allied Health Literature (CINAHL) to identify studies published in peer-reviewed journals between January 1, 2012 and December 31, 2016. Reference lists of articles identified in the search were included and were subject to the same eligibility evaluation.

### Searching and screening

We searched the health technology literature using the key terms suggested by Goundrey-Smith. All technology studies selected included the secondary search terms: “pharmacy,” “pharmacist,” or “pharmacies.” The search strategy for each database included the concepts: pharmaceutical care technologies; pharmacy. The former included the following terms derived from Goundrey-Smith: “mobile health,” “monitoring technologies,” “smart pump,” “smart packaging” and “telechealth.” Other keywords associated with telehealth (suggested on EBSCO) were included: “telemonitoring,” “teleconsultation,” “telecare,” and “telemedicine.” Goundrey-Smith also discusses the potential of mobile phone apps having an impact on pharmacy practice. A range of search terms (suggested by EBSCO) associated with mobile technology were included: “mHealth,” “m-health,” “mobile health,” and “medical apps.” “Health technology,” and “health information technology” were specified as umbrella terms for other technologies not included in our list. For our secondary search concept, “pharmacy” was defined as “pharmacy,” “pharmacies,” or “pharmacist.” Titles and abstracts were screened to remove studies that were clearly irrelevant to the aim of the review (by IG and DB). The full texts of the remaining original studies were then examined to determine eligibility.

### Study selection

Article titles and abstracts were screened independently by two different authors, IG and DB against the inclusion and exclusion criteria. Search results were imported into a Microsoft Excel and manually screened by these authors to remove any duplicates. Article titles were shortlisted for a detailed review of their abstracts. Abstracts that seemed appropriate were then screened for a full-text review. A quality assessment was performed on the articles eligible for the review. Throughout this process, we focused on articles published in English that were evaluations in the categories of mobile health, telehealth, smart pumps and monitoring technologies (the “comparisons”) of pharmaceutical care technologies (the “interventions”) tested on patients and staff (the “participants”) in a pharmacy-related setting. Study protocols and reviews were not included.

### Data collection process

A data extraction table was created to collect relevant information for each included study. The principal investigator extracted data using a data extraction form (table format) that was verified by the second reviewer. The extracted data included the name of the author, year of publication, journal, place, methodology, sample size, study design, intended market, primary outcome and study type.

### Quality analysis

We analyzed the quality (the “outcome”) of this work and reported the evaluation methods used (the “study design”). To assess the quality of each paper, scoring was undertaken using the criteria for evaluating primary research papers developed by Kmet et al. For quantitative studies, a series of 14 questions are used to assess quality. These include questions related to study design, methods of subject selection, random allocation procedures, blinding of investigators, blinding of subjects, outcome measures, sample size, estimates of variance, confounding, reporting of results and the evidence base for the conclusion. Each question is scored using the following options: Yes (2 points), Partial (1 point), No (0 points), and N/A. The equation for estimating the quality scores is: 28 minus (number of N/A × 2). The figure of 28 is the maximum score possible for the 14 questions, which can be given a maximum score of 2. We calculated a quality score for 33 quantitative papers using this method and report the results below.

As they will often use different methods to their quantitative counterparts, Kmet et al. (2004) recommend a different means of evaluating the quality of qualitative studies. Instead of 14, only 10 questions are used to assess the qualitative papers. The questions assess study design, context of the study, sampling strategy, data collection and methods of data analysis. Each question is scored using the following options: Yes (2), Partial (1), and No (0). This method includes a N/A option. The equation calculates the summary score for qualitative study as follows: Total score/20. The figure of 20 is the maximum score possible for 10 questions, which can be given a maximum score of 2. We calculated a quality score for the seven qualitative studies sampled.

The quality score system was developed to define a minimum threshold for the inclusion of studies in systematic reviews. Kmet et al. (2004) suggest two cut-points for article inclusion. Scores over 75% are classed as being acceptable using a “relatively conservative” quality benchmark. Scores over 55% are included using a “relatively liberal” cut-off point. In this paper, we report the number of quantitative and qualitative studies that meet the conservative and liberal criteria. However, we do not exclude papers from our analysis on this basis. Because we do not intend to analyse the effectiveness of individual interventions at a patient-level, the usual forms of bias do not apply to our review (Higgins and Green, 2008).

## Results

### Study selection and characteristics

**Figure 1:**
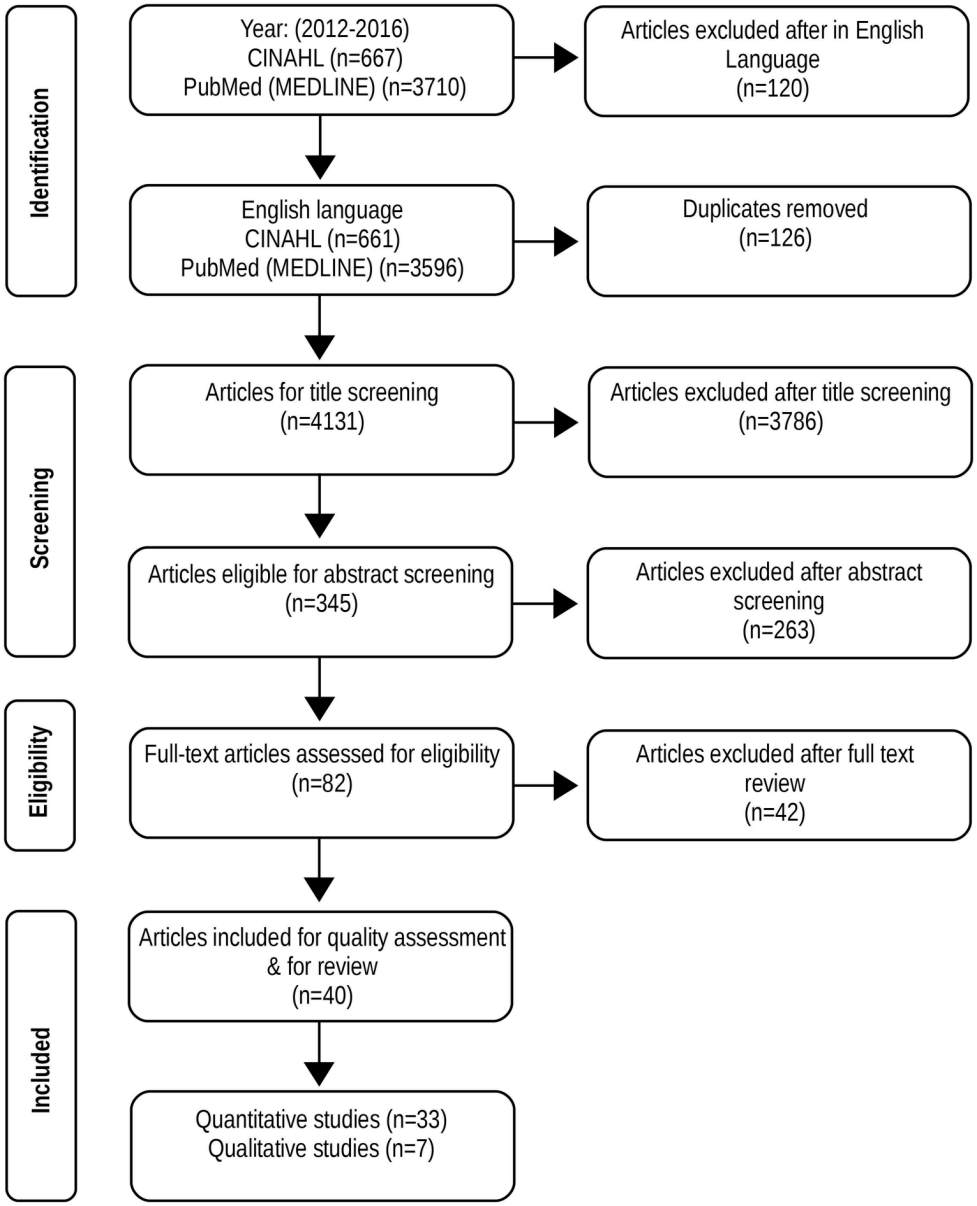
Flow diagram of scoping review search.

As Figure 1 shows, our initial search led to a total of 4,377 results (3,710 MEDLINE and 667 CINAHL). Following an analysis of publication titles, 345 papers were manually screened for abstract analysis. After reading the abstracts, 82 publications were selected for full-text assessment. The full reading resulted in 42 papers being deemed irrelevant to our review and 40 formal evaluations were finally selected.

Table 1 shows that the studies identified had the following characteristics. First, 32 out of 40 (80.00%) of the evaluations reviewed were performed in the United States, followed by three (7.50%) in the Netherlands. Second, 33 studies (82.50%) were quantitative and seven (17.50%) qualitative. Third, amongst the papers retrieved, the most common study designs employed were (with % of frequency in brackets) retrospective cohort study (24, 60.00%) and randomized controlled trials (RCTs) (9, 22.50%). Fourth, the intended setting for the technologies evaluated were: 22 primary care (55.00%); 10 hospital pharmacy (25.00%); 7 community pharmacy (17.50%); one primary care and hospital pharmacy (2.50%). Finally, out of the 40 evaluations, 33 (82.50%) reported clinical outcomes, six (15.00%) measured clinical and economic outcomes, and one (2.50%) economic only.

**Table 1:** Summary characteristics of articles included in the review (*n* = 40).

**Characteristics**	**Studies (*n*, %)**
**COUNTRY**	
Australia	(1, 2.50)
Canada	(1, 2.50)
Italy	(1, 2.50)
Spain	(1, 2.50)
UK	(1, 2.50)
Netherlands	(3, 7.50)
USA	(32, 80.00)
**METHODS**	
Qualitative	(7, 17.50)
Quantitative	(33, 82.50)
**STUDY DESIGN**	
Cross-sectional study	(1, 2.50)
Economic evaluation	(1, 2.50)
Non-randomized controlled trial	(1, 2.50)
Prospective observational study	(4, 10.00)
Randomized-controlled trial	(9, 22.50)
Retrospective cohort study	(11, 27.50)
Formative study	(13, 32.50)
**INTENDED MARKET**	
Primary care and hospital pharmacy	(1, 2.50)
Community pharmacy	(7, 17.50)
Hospital pharmacy	(10, 25.00)
Primary Care	(22, 55.00)
**OUTCOME**	
Economic	(1, 2.50)
Clinical and Economic	(6, 15.00)
Clinical	(33, 82.50)

### Results by technology category

This review found that only a handful of evaluations were published in each of the technology categories shown in Table 2. First in the ranking is “telehealth” with twenty studies (50.00%), followed by “mobile health” with nine (22.50%). Then, “monitoring technologies” produced seven (17.50%) studies. Finally, four papers (10.00%) evaluated “smart pumps.”

**Table 2:** Pharmaceutical care technologies.

**Author (year)**	**Title of study**	**Journal**	**Place**	**Methodology**	**Sample Size *n* = patients *m* = staff *p* = providers**	**Study Design**	**Intended market**	**Primary outcome**	**Study type**
**TELEHEALTH**									
Aberger et al. (2014)	Enhancing patient engagement and blood pressure management for renal transplant recipients via home electronic monitoring and web-enabled collaborative care	Telemedicine and e-Health	USA	Quantitative	*n* = 66	Retrospective cohort study	Hospital pharmacy	Blood pressure	Clinical (Positive)
Brunetti et al. (2016)	The CAPITAL study (CArdiovascular Prevention wIth Telecardiology in ApuLia): preliminary results	Journal of Cardiovascular Medicine	Italy	Quantitative	*n* = 1000	Retrospective cohort study	Community pharmacy	BMI; BP; Serum total cholesterol levels	Clinical (Negative)
Cole et al. (2012)	Rural inpatient telepharmacy consultation demonstration for after-hours medication review	Telemedicine and e-Health	USA	Quantitative	*n* = 302	Retrospective cohort study	Hospital pharmacy	Medication errors	Clinical (Positive)
Desko and Nazario (2014)	Evaluation of a clinical video telehealth pain management clinic	Journal of Palliative Care Pharmacotherapy	USA	Qualitative	*n* = 39	Prospective observational study	Primary care	Satisfaction of CVT pain management clinic	Clinical (Positive) Economic (Positive)
Fortney et al. (2013)	Practice-based versus telemedicine-based collaborative care for depression in rural federally qualified health centers: a pragmatic randomized comparative effectiveness trial	The American Journal of Psychiatry	USA	Quantitative	*n* = 364	Randomized-controlled trial	Primary care	Treatment response; Remission and changes in depression severity	Clinical (Positive)
Fortney et al. (2015)	Telemedicine-based collaborative care for posttraumatic stress disorder: a randomized clinical trial	JAMA Psychiatry	USA	Quantitative	*n* = 265	Randomized-controlled trial	Primary care	Posttraumatic Diagnostic Scale	Clinical (Positive)
Gordon et al. (2012)	Telepharmacy in a rural Alberta community cancer network	Journal of Oncology Pharmacy Practice	Canada	Qualitative	*n* = 47; *m* = 97	Formative study	Primary care	Satisfaction of telepharmacy service	Clinical (Positive)
Kooy et al. (2015)	Patients' general satisfaction with telephone counseling by pharmacists and effects on satisfaction with information and beliefs about medicines: Results from a cluster randomized trial	Patient Education and Counseling	Netherlands	Quantitative	*n* = 211	Randomized-controlled trial	Community pharmacy	Proportion of adherent patients, based on refill adherence	Clinical (Positive)
Margolis et al. (2013)	A telepharmacy intervention to improve inhaler adherence in veterans with chronic obstructive pulmonary disease	American Journal of Health-System Pharmacy	USA	Quantitative	*n* = 97	Randomized—controlled trial	Primary care	Frequency of use of inhaler	Clinical (Positive)
Martinez et al. (2013)	Implementation of a pharmacist-managed heart failure medication titration clinic	American Journal of Health-System Pharmacy	USA	Quantitative	*n* = 51	Retrospective cohort study	Primary care	% of patients in whom target ACEI, ARB, and b-blocker dosages were achieved	Clinical (Positive)
Martinez et al. (2013)	Implementation of a pharmacist-managed heart failure medication titration clinic	American Journal of Health-System Pharmacy	USA	Quantitative	*n* = 51	Retrospective cohort study	Primary care	% of patients in whom target ACEI, ARB, and b-blocker dosages were achieved	Clinical (Positive)
McFarland et al. (2012)	Use of home telehealth monitoring with active medication therapy management by clinical pharmacists in veterans with poorly controlled type 2 diabetes mellitus	Pharmacotherapy	USA	Quantitative	*n* = 103	Non-randomized Controlled trial	Primary care	Change in A1C from baseline to 6 months	Clinical (Negative)
Owsley et al. (2015)	Diabetes eye screening in urban settings serving minority populations detection of diabetic retinopathy and other ocular findings using telemedicine	JAMA Ophthalmology	USA	Quantitative	*n* = 1894	Cross-sectional study	Community pharmacy	% of type and % of diabetic retinopathy detection	Clinical (Positive)
Philip et al. (2015)	Expansion of clinical pharmacy through increased use of outpatient pharmacists for anticoagulation services	American Journal of Health-System Pharmacy	USA	Quantitative	*n* = 201	Retrospective cohort study	Hospital pharmacy	International normalized ratio; Patient clinic visits	Clinical (Positive)
Schneider (2013)	Evaluating the impact of telepharmacy	American Journal of Health-System Pharmacy	USA	Quantitative	*p* = 3	Formative study	Hospital pharmacy	Medication errors	Clinical (Positive) Economic (Positive)
Shane-McWhorter et al. (2015)	Pharmacist-provided diabetes management and education via a telemonitoring program	Journal of the American Pharmacists Association	USA	Quantitative	*n* = 150	Prospective observational study	Primary care	Change in A1C levels measured at baseline and at conclusion of the monitoring period	Clinical (Positive)
Singh et al. (2015)	Implementation and outcomes of a pharmacist-managed clinical video telehealth anticoagulation clinic	American Journal of Health-System Pharmacy	USA	Quantitative	*n* = 38	Formative study	Primary care	International normalized ratio	Clinical (Positive)
Verbosky et al. (2016)	Implementation and evaluation of diabetes management via clinical video telehealth	Diabetes Care	USA	Qualitative	*n* = 14	Formative study	Primary care	Patient satisfaction with clinical video telehealth program	Clinical Economic (Positive, Positive)
Wang et al. (2012)	Economic evaluation of telephone self-management interventions for blood pressure control	American Heart journal	USA	Quantitative	*n* = 591	Economic evaluation	Primary care	Intervention costs; Home BP monitoring	Economic (Negative)
Young et al. (2012)	Patient And phaRmacist Telephonic Encounters (PARTE) in an underserved rural patient population with asthma: Results of a pilot study	Telemedicine and e-Health	USA	Quantitative	*n* = 98	Randomized controlled trial	Primary care	Asthma Control Test; Participants attitudes with intervention	Clinical (Positive)
**Mobile Health**									
Andrus et al. (2015)	Accuracy of pharmacy benefit manager medication formularies in an electronic health record system and the Epocrates mobile application	Journal of Managed Care and Specialty Pharmacy	USA	Quantitative	1529 medical records	Retrospective cohort study	Hospital pharmacy	Accuracy of formulary information in EHR and Epocrates app	Clinical (Negative)
Burk et al. (2013)	Medication-use evaluation with a Web application	American Journal of Health-System Pharmacy	USA	Qualitative	*p* = 147	Formative study	Primary care	Medication use evaluation tracker interventions	Clinical (Positive)
DiDonato et al. (2015)	Community pharmacy patient perceptions of a pharmacy-initiated mobile technology app to improve adherence	International Journal of Pharmacy Practice	USA	Qualitative	*p* = 435	Formative study	Community pharmacy	Medication adherence; Patient opinion pharmacist driven app	Clinical (Positive)
Foreman et al. (2012)	Impact of a text messaging pilot program on patient medication adherence	Clinical Therapeutics	USA	Quantitative	*n* = 580	Retrospective cohort study	Community pharmacy	Medication adherence	Clinical (Positive)
Gatwood et al. (2016)	The impact of tailored text messages on health beliefs and medication adherence in adults with diabetes: A randomized pilot study	Research in Social and Administrative Pharmacy	USA	Quantitative	*n* = 75	Formative study	Primary care	Medication adherence	Clinical (Negative)
Gustafson et al. (2012)	The effects of combining web-based eHealth with telephone nurse case management for pediatric asthma control: A randomized controlled trial	Journal of Medical Internet Research	USA	Quantitative	*n* = 301	Randomized—controlled trial	Primary care	Medication adherence, symptom free days, asthma control questionnaire	Clinical (Negative)
Sarzynski et al. (2017)	Beta Testing a Novel Smartphone Application to Improve Medication Adherence	Telemedicine Journal and e-Health	USA	Quantitative	*n* = 8	Formative study	Community pharmacy	Accuracy of auto-populated medication dosing instructions; Acceptability of the user interface; Patient adherence	Clinical (Positive)
Snuggs et al. (2012)	Using text messaging to prevent relapse to smoking: intervention development, practicability and client reactions addiction	Addiction	UK	Quantitative	*n* = 202	Retrospective cohort study	Primary care	Response to interactive messages and requests for medication	Clinical (Positive)
Wilcox et al. (2016)	Interactive tools for inpatient medication tracking: a multi-phase study with cardiothoracic surgery patients	Journal of the American Medical Informatics Association	USA	Qualitative	*n* = 12	Formative study	Primary care	Patient and pharmacist responses to medication tracking app	Clinical (Positive)
Bender et al. (2015)	Pragmatic trial of health care technologies to improve adherence to pediatric asthma treatment: a randomized clinical trial	JAMA pediatrics	USA	Quantitative	*p* = 899	Randomized-controlled trial	Primary care and hospital pharmacy	Medication possession ratio	Clinical (Positive)
Chan et al. (2015)	The effect of an electronic monitoring device with audiovisual reminder function on adherence to inhaled corticosteroids and school attendance in children with asthma: a randomized controlled trial	Lancet Respiratory Medicine	Australia	Quantitative	*n* = 220	Randomized—controlled trial	Primary care	Adherence to preventive inhaled corticosteroids and number of days absent from school for any reason	Clinical (Positive)
**Monitoring Technologies**									
Migliozzi et al. (2015)	Achieving blood pressure control among renal transplant recipients by integrating electronic health technology and clinical pharmacy services	American Journal of Health-system Pharmacy	USA	Quantitative	*n* = 37	Formative study	Hospital pharmacy	Blood pressure	Clinical (Positive)
Sayner et al. (2015)	Accuracy of patient-reported adherence to glaucoma medications on a visual analog scale compared with electronic monitors	Clinical Therapeutics	USA	Quantitative	*n* = 240	Retrospective cohort study	Primary care	Self-reported medication adherence; Returned MEMS monitors	Clinical (Negative)
Van Onzenoort et al. (2012)	Determining the feasibility of objective adherence measurement with blister packaging smart technology	American Journal of Health-System Pharmacy	Netherlands	Quantitative	*n* = 115	Formative study	Community pharmacy	Smart blister equipped medication card	Clinical (Positive)
Vasbinder et al. (2013)	The association of ethnicity with electronically measured adherence to inhaled corticosteroids in children	European Journal of Clinical Pharmacology	Netherlands	Quantitative	*n* = 90	Prospective observational study	Primary care	Electronic adherence measurements	Clinical (Negative)
Zullig et al. (2014)	A health literacy pilot intervention to improve medication adherence using Meducation® technology	Patient Education and Counseling	USA	Quantitative	*n* = 23	Formative study	Primary care	Medication adherence; Blood pressure; Body weight	Clinical (Positive)
**Smart Pumps**									
Gerhart et al. (2013)	Advancing medication infusion safety through the clinical integration of technology	Hospital Practice	USA	Quantitative	*p* = 13	Retrospective cohort study	Hospital pharmacy	Medication error; Compliance rate; Facility costs	Clinical (Positive) Economic (Positive)
Harding (2012)	Increasing the use of “smart” pump drug libraries by nurses: A continuous quality improvement project	American Journal of Nursing	USA	Quantitative	System evaluation	Retrospective cohort study	Hospital pharmacy	Medication errors	Clinical (Positive) Economic (Positive)
Kennerly et al. (2012)	Implementing smart pumps for epidural infusions in an academic medical center	American Journal of Health-System Pharmacy	USA	Qualitative	*n* = 77	Formative study	Hospital pharmacy	Opinions before and after implementation smart pump	Clinical (Positive)
Manrique-Rodríguez et al. (2013)	Impact of implementing smart infusion pumps in a pediatric intensive care unit	American Journal of Health-System Pharmacy	Spain	Quantitative	*n* = 500	Prospective observational study	Hospital pharmacy	Medication errors	Clinical (Positive) Economic (Positive)

### Telehealth

Table 2 shows that 20 studies focused on telehealth. They include evaluations of telehealth, telemedicine and telemonitoring. Seventeen of the 20 studies sampled were American, with one study each from Italy, Canada and the Netherlands. Three were qualitative and 17 were quantitative. Nineteen sampled patients to test their technologies; one sampled staff. Retrospective cohort study was the most commonly used study design. Six out of eight studies published were conducted in a primary care setting, one took place in community pharmacy and the other in hospital pharmacy. All studies specified a primary outcome. Six undertook a clinical evaluation, one conducted a clinical and economic evaluation and one performed an economic evaluation only. All six of the clinical evaluations produced positive results. The joint clinical and economic study was positive in both dimensions and the stand alone economic study was negative.

### Mobile health

Table 2 shows that nine evaluations focused on mobile health technologies. However, eight out of the nine studies were conducted in the USA and one in the UK. Of the studies published, three were qualitative and six were quantitative. Six out of nine studies recruited patients to test their technologies. Two studies used providers and another used patient medical records. Three studies employed a retrospective cohort design, five employed a formative design and one was a RCT. Five studies were undertaken in a primary care setting, three in community pharmacy and one in hospital pharmacy. All studies specified a primary outcome and performed a clinical evaluation. No economic or costing work was undertaken. Four of the nine studies had negative results.

### Monitoring technologies

Table 2 shows that seven studies focused on monitoring technologies, which includes smart packaging. All seven were quantitative and used patients to test their technologies. Out of these, three were formative, two were RCTs, one was a prospective observational study and one was a retrospective cohort study. Four studies were conducted in primary care alone, one in community pharmacy, one in hospital pharmacy and one jointly in primary care and hospital pharmacy. All the studies specified a primary outcome and performed a clinical evaluation of their chosen technology. No economic or costing studies were performed. Five of the seven studies produced positive results.

### Smart pumps

Table 2 shows that four studies evaluated smart pumps. One study was qualitative and the remaining three were quantitative. Two studies sampled patients to test their technologies. One evaluation focused on providers and the other performed a system evaluation. A retrospective cohort design was used for two studies. One was a formative evaluation and another was a prospective observational study. All studies were conducted in a hospital pharmacy setting and stated a primary outcome. Two undertook clinical evaluations. Two conducted an economic evaluation alongside clinical studies. All evaluations produced positive findings.

### Quality assessment

Table 3 shows the quality scores for both quantitative and qualitative studies. In terms of quality, 12 (30.00%) quantitative studies and no qualitative study met the Kmet et al. (22) criteria for “relatively conservative” quality. Using the lower “relatively liberal” benchmark, 27 (67.50%) quantitative studies and four (10.00%) qualitative study passed. The table also shows the quality score for each of the 33 quantitative studies, which was calculated using the Kmet et al. framework. The quantitative summary scores vary from 39 to 88. Applying the conservative benchmark of 75%, 21 (63.64%) out of the 33 quantitative studies were of insufficient quality. If the standard is dropped, using the 55% cut-off only six (18.18%) studies fail to meet the required benchmark. Therefore, whether 15 (45.45%) studies have acceptable quality depends upon which of the two cut-offs is used.

**Table 3:** Quality analysis table for quantitative and qualitative studies.

**Author**	**Objective and Study design**	**Subjects selection**	**Random allocation and blinding**	**Outcome**	**Sample size and analytical methods**	**Results supported by conclusion**	**Total sum**	**Total possible sum**	**Summary Score (%)**	**Liberal threshold (55 ≤ %)**	**Conservative threshold (75 ≤ %)**
**QUANTITATIVE STUDIES**											
**Telehealth**											
Aberger et al. (2014)	2	1	0	2	5	3	13	28	46	0	0
Brunetti et al. (2016)	2	3	0	1	7	2	15	22	47	0	0
Cole et al. (2012)	4	3	0	1	3	4	15	22	68	1	0
Fortney et al. (2013)	4	4	2	2	7	1	20	24	83	1	1
Fortney et al. (2015)	4	4	3	2	6	3	22	26	85	1	1
Kooy et al. (2015)	4	2	2	2	8	3	21	24	88	1	1
Margolis et al. (2012)	4	2	5	1	5	2	19	28	68	1	0
Margolis et al. (2013)	4	2	4	1	2	2	15	28	54	0	0
Martinez et al. (2013)	3	3	0	2	4	2	14	22	64	1	0
McFarland et al. (2012)	4	4	0	2	6	3	19	22	86	1	1
Owsley et al. (2015)	4	3	0	2	5	3	17	22	77	1	1
Philip et al. (2015)	4	3	4	1	4	2	18	28	64	1	0
Schneider (2013)	4	3	0	1	4	4	16	22	73	1	0
Shane-McWhorter et al. (2015)	4	3	0	2	7	4	20	24	83	1	1
Singh et al. (2015)	3	3	0	1	5	2	14	22	64	1	0
Wang et al. (2012)	3	2	2	2	5	3	17	28	61	1	0
Young et al. (2012)	4	4	3	1	6	2	20	28	71	1	1
**Mobile health**											
Andrus et al. (2015)	3	0	0	2	2	4	11	18	61	1	0
Foreman et al. (2012)	4	4	1	2	5	2	18	28	64	1	0
Gatwood et al. (2016)	4	4	2	2	7	3	22	28	79	1	1
Gustafson et al. (2012)	4	4	2	2	5	3	20	28	71	1	0
Sarzynski et al. (2017)	4	3	0	2	4	3	16	22	73	1	1
Snuggs et al. (2012)	3	3	0	2	5	3	16	22	73	1	0
**Monitoring Technologies**											
Bender et al. (2015)	4	4	1	2	6	3	20	24	83	1	1
Chan et al. (2015)	4	4	4	2	7	3	24	28	86	1	1
Migliozzi et al. (2015)	3	3	0	1	2	2	11	22	50	0	0
Sayner et al. (2015)	4	3	2	1	5	2	17	28	61	1	0
Van Onzenoort et al. (2012)	3	1	0	1	5	3	13	20	65	1	0
Vasbinder et al. (2013)	4	4	0	2	7	2	19	22	86	1	1
Zullig et al. (2014)	3	4	1	1	3	2	14	24	58	1	0
**Smart Pumps**											
Gerhart et al. (2013)	1	0	0	1	3	3	8	18	44	0	0
Harding (2012)	2	0	0	1	2	2	7	18	39	0	0
Manrique-Rodríguez et al. (2013)	4	1	0	1	4	2	12	22	55	1	0
**Author**	**Objective and study design**	**Framework and sampling**	**Data collection and Analysis**	**Verification and conclusion**	**Total sum**	**Total possible sum**	**Summary score (%)**		**Liberal threshold (55 ≤ %)**		**Conservative threshold (75 ≤ %)**
**QUALITATIVE STUDIES**											
**Telehealth**											
Desko and Nazario (2014)	5	1	3	3	12	20	60	1		0	
Gordon et al. (2012)	4	2	1	2	9	20	45	0		0	
Verbosky et al. (2016)	3	0	0	1	4	20	20	0		0	
**Mobile Health**											
Burk et al. (2013)	5	2	2	1	10	20	50	0		0	
DiDonato et al. (2015)	5	3	3	3	14	20	70	1		0	
Wilcox et al. (2016)	6	3	4	1	14	20	70	1		0	
**Smart Pumps**											
Kennerly et al. (2012)	4	2	4	2	12	20	60	1		0	

Quality scores were also calculated for the seven qualitative papers identified, which produced a range of 20–70. As Table 3 shows, using the conservative criteria only, no study was of sufficient quality. Under the liberal criteria, four (57.14%) papers managed to reach the required standard. Therefore, a switch of criteria results in a difference of four (57.14%) papers being acceptable in quality terms.

### Study quality and design

Table 4 shows average quality scores by study design and technology categories. The following quality scores were obtained for quantitative studies: cross-sectional (77.00%); economic evaluation (61.00%); formative (66.00%); non-randomized controlled trial (86.00%); prospective observational (74.67%); RCT (86.13%); retrospective cohort (70.11%). The qualitative scores are: formative (52.50%); prospective observational (60.00%). The sub-totals show that the average quality score for quantitative studies is 64.97% and for qualitative is 53.57%. The results also show quality scores by technology: mobile health (67.89%); monitoring technologies (69.86%); smart pumps (49.50%); telehealth (65.35%).

**Table 4:** Average percentage quality score by study design and technology.

	**Mobile health (%)**	**Monitoring technologies (%)**	**Smart pump (%)**	**Telehealth (%)**	**Study design average quality score (%)**
**Quantitative studies**					
Cross-sectional study	–	–	–	77	77.00
Economic evaluation	–	–	–	61	61.00
Formative study	76	57.67	–	43.84	66.00^a^
Non-randomized controlled trial	–	–	–	86	86.00
Prospective observational study	–	86	55	83	74.67^b^
Randomized-controlled trial	71	84.5	-	74.83	86.13^c^
Retrospective cohort study	66	61	41.5	57.8	70.11^d^
Average of quantitative studies per technology category	70.17	69.86	46	69.53	–
**Qualitative studies**					
Formative study	63.33	–	60	32.5	52.50^e^
Prospective observational study	–	–	–	60	60
Average of qualitative studies per technology category	63.33	–	60	41.67	–
Average quality score of qualitative and quantitative studies per technology category	67.89	69.86	49.50	65.35	–
Sub-total of quantitative studies	64.97				
Sub-total of qualitative studies	53.57				
Sub-total of all studies	62.98				

a *(50+58+64+65+73+73+79)/7 = 66.00*.

b *(55+83+86)/3 = 74.67*.

c *(54+68+71+71+83+83+85+86+88)/8 = 86.13*.

d *(39+44+46+47+61+61+64+64+64+68+73)/9 = 70.11*.

e *(20+45+50+60+70+70)/6 = 52.50*.

## Discussion

Without sufficient evidence, novel technologies may fail to be adopted in a pharmacy-related setting (Siska and Tribble, 2011). The review presented here suggests that, worldwide, few evaluations of pharmaceutical care technologies are currently being undertaken in such a place. With only 40 relevant studies being identified over a 5-year period, the first finding of our review is that pharmacy-related evaluations of new technologies are relatively rare. This number is small, given the number of new technologies that appear each year. However, in interpreting this data, we must add the caveat that many of the health technologies launched each year may be evaluated for general use in any health setting, which covers pharmacy by default. For instance, Dayer et al. (2013) reviews the potential benefits of Smartphone medication adherence apps to patients and providers, but does not undertake this work solely with pharmacist input or in a pharmacy-related setting. Therefore, their paper did not appear in our search. As this suggests, pharmacy specific evaluations may not be needed for many new health technologies. Our search is, consequently, narrow in its focus. Even if these caveats are considered, the numbers of evaluations identified in this review are still relatively low.

Whilst it is useful to speculate about the relative number of papers published in this area, the main focus of this review is study quality and design. Using the Kmet et al. (2004) scoring system, we found a wide dispersion in the quality of papers published. Only 30.00% of quantitative studies met the higher, conservative benchmark. In comparison, no qualitative studies achieved this quality standard. In a sector familiar with high-quality evidence accompanying the launch of new pharmaceutical products, new technologies with low quality evidence may struggle to be adopted. Therefore, our third finding is that the quality of most of evaluations we reviewed is lacking when compared to objective criteria. As the Kmet et al. (2004) scoring system is primarily based upon the quality of study design, we suggest that inadequate study design is the cause of these lower quality scores. This view is support by the data presented in Table 4.

We classify pharmaceutical care technologies as including mobile health, telehealth, smart pumps and monitoring technologies. These technologies are pharmacy-enabling because they extend the knowledge and the capabilities of pharmacists, enabling them to create better outcomes for patients. This category suggests that pharmaceutical care activities may be enhanced by new health technologies, as well as an extension of professional skills and roles (Lapão et al., 2013). Of the 40 papers we identified, 33 were quantitative and 7 were qualitative. The average quality score in the former group is (2144/33) = 64.97% and (375/7) = 53.57% in the latter. Both these average scores are below the Kmet et al. relatively conservative quality benchmark. We discuss the evaluations in these classes below.

Telehealth is an enabling-technology for patients (Dewsbury and Ballard, 2012). We found 20 studies relevant to pharmacy. Margolis evaluates telemonitoring for home blood pressure in hypertension care (Margolis et al., 2012). Similarly, Wang performs an economic evaluation of telephone self-management for blood pressure control (Wang et al., 2012). Margolis examines the use of telepharmacy to improve inhaler adherence in veterans with COPD (Margolis et al., 2013). Martinez studies the implementation of a pharmacist-led heart titration clinic that employs telemonitoring for the daily monitoring of patient body weight (Martinez et al., 2013). Philip analyses the expansion of clinical pharmacy through increased use of outpatient pharmacists for anticoagulation services (Philip et al., 2015). Verbosky assesses the implementation of diabetes management via telehealth, whilst Owsley evaluates telemedicine for diabetic eye screening (Owsley et al., 2015; Verbosky et al., 2016). Singh studies the implementation and benefits of a pharmacist-managed telehealth anticoagulation clinic (Singh et al., 2015). Aberger evaluates a telehealth system that incorporates home blood pressure monitoring (Aberger et al., 2014). Brunetti describes the CAPITAL study, which focuses on cardiovascular prevention with telecardiology (Brunetti et al., 2016). Cole examines inpatient telepharmacy consultation and medication errors (Cole et al., 2012). Desko evaluates a clinical video telehealth pain management clinic (Desko and Nazario, 2014). Gordon investigates a telepharmacy initiative for cancer patients (Gordon et al., 2012). Fortney evaluates telemedicine collaborative care for depression (Fortney et al., 2015). Fortney analyses telemedicine-based collaborative care for post-traumatic stress disorder (Fortney et al., 2013). Kooy examines patient satisfaction with telephone counseling by pharmacists and medication refill adherence (Kooy et al., 2015). McFarland examines the use of home telehealth monitoring by clinical pharmacists with type 2 diabetic patients (McFarland et al., 2012). Schneider evaluates the impact of telepharmacy through medication errors (Schneider, 2013). Shane-McWhorter analyses a pharmacist-provided diabetes management telemonitoring program (Shane-McWhorter et al., 2015). Young investigates patient and pharmacist telephone encounters with asthma patients (Young et al., 2012).

The above studies suggest that telehealth technologies employed in a pharmacy context focuses on communication between pharmacists and patients. This is very different to telecare as monitoring devices (such as pendant alarms) located in patients' own homes (Steventon et al., 2013; Roulstone, 2016). Therefore, greater clarity on the types and the benefits of telehealth, telecare and telepharmacy would be useful for guiding future pharmacy research and practice, particularly as the use of this category of technology raises practical and ethical issues (Mitka, 2013; Mort et al., 2013). Given the personalized nature of this technology, all of the 20 studies identified recruited patients to test their innovative technologies using a range of study designs. The average quality scores for the studies were: formative evaluations (51%); RCTs (75%); retrospective cohort studies (58%); cross-sectional study (77%); economic evaluation (61%); prospective observational study (72%); non-randomized controlled trial (86%). These results imply that the quality standards achieved in this category of evaluation were variable, with implications for the trustworthiness of the evidence generated.

Our review identified nine papers evaluating mobile health technologies (Steinhubl et al., 2015). Gustafson examines the effects of a patient support system that includes monthly phone calls carried out by pharmacists and other health care professionals (Gustafson et al., 2012). Foreman assesses the impact of text messaging to improve patient medication adherence, whilst Snuggs analyses the use of texting to prevent relapses in smoking (Foreman et al., 2012; Snuggs et al., 2012). Burk examines a web-based app for medication use in a veteran's healthcare system (Burk et al., 2013). Andrus assesses the accuracy of formulary information in both EHR and the Epocrates mobile drug database application, used for making medication selection decisions (Andrus et al., 2015). Wilcox designed a personal health record application called “myNYP inpatient” and assesses its usefulness for patients (Wilcox et al., 2016). DiDonato evaluates patient perceptions of a pharmacy mobile app for patient adherence (DiDonato et al., 2015). Gatwood studies the impact of tailored text messages on medication usage amongst diabetic patients (Gatwood et al., 2016). Sarzynski uses beta-testing on a smartphone application for medication adherence (Sarzynski et al., 2017).

Compared to the large number of health apps launched annually, the handful of studies identified suggests that very few mobile health technologies are reviewed formally in a pharmacy setting. The absence of such scientific evaluations may be evidence of a “valley of death” between product availability and use in pharmacy practice (Wessner, 2005; Páez-Avilés et al., 2015). For a profession keen on creating new roles and opportunities, the failure to adopt new mobile technologies as part of the pharmacist's tool-kit may limit continued professional growth (Miranda et al., 2014; Ventola, 2014). Of the studies published, three studies employ a retrospective cohort design, five employ a formative design and one is a RCT. Their average quality scores are 66, 69, and 71%, retrospectively. These scores suggest that improved study designs could generate higher quality evidence in this category.

Seven studies were identified that evaluate monitoring technologies in a pharmacy context (Speedie et al., 2008; Fox et al., 2011). Van Onzenoort studies smart blister-pack technology for checking patient adherence (Van Onzenoort et al., 2012). Vasbinder assesses the relationship between ethnicity and electronically measured adherence to inhaled corticosteroids amongst children (Vasbinder et al., 2013). Zullig pilots a medication calendar that incorporates reminders using Meducation^®;^ technology (Zullig et al., 2014). Chan evaluates the effects of using electronic monitoring devices for adherence to inhaled corticosteroids, whilst Migliozzi looks at controlling blood pressure using home monitoring (Chan et al., 2015; Migliozzi et al., 2015). Sayner compares self-reported adherence data with information collected form a medication event monitoring system (Sayner et al., 2015). Finally, Bender evaluated the use of a novel technology to improve the adherence of pediatric asthmatic patients (Bender et al., 2015). Combined, these studies suggest that monitoring technologies have an important role to play in enabling pharmacists to achieve better outcomes for patients, particularly in relation to medication adherence (Bosworth et al., 2011). However, the volume of evidence generated is low given the potential benefits offered by innovations in this category. Although all seven evaluations are quantitative and involve patients, study designs are not uniform: three are formative (50, 58, 65%); two are RCTs (83, 86%); one is a prospective observational study (86%); one is a retrospective cohort study (61%). Their quality scores are shown in the proceeding brackets.

Smart pumps are electronic infusion devices that have the potential to reduce intravenous drug administration errors (Franklin, 2017). Our review found four published studies. Harding examines the use of smart pump drug libraries where continuing education is provided for nurses and pharmacists (Harding, 2012). Kennerly studies smart pumps for epidural infusions, whilst Manrique-Rodriguez evaluates the impact of introducing smart pumps into a pediatric intensive care unit (Kennerly et al., 2012; Manrique-Rodríguez et al., 2013). Finally, Gerhart combines the safety features of infusion pumps with software that shows live infusion data (Gerhart et al., 2013). Smart pumps are an important enabling technology for pharmacy practice. However, our review creates a mixed message of whether more studies are required. In our search terms, we specified that evaluations must be performed in a pharmacy-related setting. However, this category of technology may also be identified as medicines or guidelines related, particularly in the case of medication libraries containing dosing guidelines, concentrations, clinical advice and the like. We conclude that smart pumps are a vital technology in pharmacy practice, but we have identified few evaluations in this category using our key terms. In terms of study design, two evaluations employ a retrospective cohort design (39%, 44%), one is a formative evaluation (60%) and another is a prospective observational study (55%). Relevant quality scores are in the brackets shown.

First, health technology is a diverse field, which covers many thousands of new innovations launched each year. We identified a new class of studies, which we call “pharmaceutical care technologies.” This division is innovative. More research is, therefore, required to ascertain whether this novel categorization is useful for pharmacy practice and research. Second, our review focuses on technologies that enable pharmacists and pharmacy practice. However, many technologies are designed to enable patients (not their practitioners). Our approach may be orthogonal to the intentions of many device manufacturers, particularly when considering personalized health apps. Therefore, further research is required to examine the differences between practitioner- and patient-enabling technologies in a pharmacy-related setting. Finally, we focus on technologies suggested by Goundrey-Smith. We have not searched for all possible health technologies that could be adopted in a pharmacy-related setting. Notably, “wearables” and the “internet of things” were not included in our review. However, an initial search identified no relevant studies in the time-period we examined. As these new technologies grow in influence, more comprehensive reviews of the literature will be required.

## Implications of the review for pharmacy practice

The technological innovations discussed have the potential to improve pharmacy practice related tasks and functions. In addition, evidence from included studies suggested that the systems could be improved through innovation involving technology. Pharmacists should not assume that all new health technologies are automatically good for patients (Draper and Sorell, 2013). Therefore, this review suggests that the process of evaluating new health technologies should be continuous and be of the highest research quality possible (Catwell and Sheikh, 2009; Wapner, 2016). Not only will high quality evidence support the rational adoption of new pharmacy-enabling technologies, but better evidence may help avoid some of the unintended problems associated with the use of novel innovations (Patterson et al., 2002; Koppel et al., 2008). To secure the future of the profession, as technology evolves, pharmacy practice must evolve, too (Baines, 2008).

## Conclusions

This paper reviews the quality and the design of studies published in peer-reviewed journals that report scientific evaluations of new health technologies tested in a pharmacy-related setting. The results suggest that, worldwide, few evaluations of pharmaceutical care technologies are currently being undertaken. With only 40 relevant studies being identified, the first finding of our review is that pharmacy-based evaluations of new technologies are relatively rare (not many have had a pharmacy focus). Our second finding is that the quality of most of the evaluations we reviewed was lacking when compared to objective criteria. As the Kmet et al. scoring system is primarily based upon the quality of study design, we can conclude that inadequate study design may be a significant cause of these lower quality scores. Despite the improvements in technology, there is limited evidence on how this translates to real settings and to consumer satisfaction. Most technology driven systems required significant funding and support, particularly those involving latest technology. Rigorous comparative studies are needed to evaluate the effectiveness of different technologies.

## Author contributions

IG and DB conceptualized the idea. IG developed the search strategy, conducted the searches, screened, selected and performed a quality scoring of the studies. DB was also involved in the title screening as the second reviewer. AH and AK were involved in drafting the manuscript and in the quality scoring of the studies. ZB, SH, and PS were also involved in revising the manuscript, checking the study selection process, quality scoring of the studies and tables. All authors contributed, read and approved the final version of the manuscript.

### Conflict of interest statement

The authors declare that the research was conducted in the absence of any commercial or financial relationships that could be construed as a potential conflict of interest.
